# High Bone Mineral Density of the Lumbar Spine Is Positively Associated with Breast Cancer

**DOI:** 10.1155/2019/8010356

**Published:** 2019-05-23

**Authors:** Larissa Vaz Gonçalves, Karine Anusca Martins, Jordana Carolina Marques Godinho-Mota, Raquel Machado Schincaglia, Ana Luisa Lima Sousa, Ruffo Freitas-Junior

**Affiliations:** ^1^Graduate Program in Health Sciences, Faculty of Medicine, Federal University of Goiás, Brazil; ^2^Advanced Breast Diagnostic Center, Mastology Program, Hospital das Clínicas, Federal University of Goiás, Brazil; ^3^Postgraduate Program in Nutrition and Health, Faculty of Nutrition, Federal University of Goiás, Brazil

## Abstract

**Objective:**

The objective of this study was to verify possible associations between bone mineral density (BMD) and breast cancer in recently diagnosed women in the Brazilian Mid-west region, considering the menopausal status of patients.

**Methods:**

A case-control study was conducted with 142 cases of breast cancer and 234 controls matched by for age, body mass index (BMI), and menopausal status (pre- and postmenopause), performed in a university hospital in the Brazilian Mid-west. Lumbar spine (L1–L4), femoral neck, and total femur BMD were measured by the dual-energy X-ray absorptiometry (DXA) method. For association, a logistic regression analysis was used.

**Results:**

Women in the highest lumbar spine BMD quartile presented had a higher chance of developing breast cancer (OR = 2.31; 1.02–5.25;* p* = 0.045), after adjusting for the confounding variables. Nonetheless, there were no statistically significant differences in the association between pre- and postmenopause in that quartile and breast cancer.

**Conclusions:**

High lumbar spine BMD was positively associated with breast cancer in the total sample. In evaluating the BMD of the femoral neck and total femur, such an association was not observed.

## 1. Background

Bone and breast tissues are estrogen-responsive [1–5]. Estrogen exerts a protective effect on bone health, aiding in the regulation of metabolism while playing a role in its maintenance [1–3]. Estrogen receptors are found on osteoblasts, the cells responsible for bone formation, and on osteoclasts, the cells responsible for bone resorption [1–3]. On the other hand, estrogen participates in the mammary cancer due to physiological stimulation on the mammary glands through the mitotic activity involved in the growth of this epithelium [4, 5].

After menopause, ovarian failure results in a significant estrogen reduction associated with rapid bone loss, which can lead to osteoporosis; consequently, fractures and falls can occur due to bone fragility, which is also a result of aging [6, 7]. Both osteoporosis and breast cancer are serious health problems, with a negative impact on the quality of life of women [7, 8]. However, they are inversely related: women with vertebral fractures related to osteoporosis have a 62.0% lower risk of breast cancer [9].

Bone mineral density (BMD) may remain high during menopause, which may be a risk for breast neoplasia since it causes a higher concentration of estrogen during menstrual phase. This association was observed in a few studies, which found high BMD in patients with breast cancer after menopause [10–14]. However, this association has been questioned in other studies that have shown an inconsistency in the relationship between bone mass and breast cancer [15, 16].

Considering BMD is an intermediate marker of estrogen exposure during the life of a woman and that the only studies identified in Brazilian women were carried out after treatment and/or in survivors and not with newly diagnosed breast cancer, [17–19], the aim of this study was to verify associations between bone mineral density and breast cancer in newly diagnosed women in the Brazilian Midwest in both pre- and postmenopausal status.

## 2. Methods

### 2.1. Study Design and Population

A case-control study with women who were newly diagnosed with breast cancer at a university hospital in the Central-west of Brazil was performed, with data collected between August 2014 and September 2017.

The study included women between the ages of 30 and 80 years old. The cancer group was composed of patients newly diagnosed with breast cancer (stages IA-III), at most two weeks after confirmation by anatomopathological report, who had not begun chemotherapy and/or hormone therapy. In the control group, volunteers attended outpatient clinics at the university hospital other than mastology and were professionals and students from the various courses of the Federal University of Goiás. All volunteers in the control group had no previous or current diagnosis of any malignant neoplasm and had no malignant changes in mammography or gynecological examinations in the last year, and all fit the established pairing with patients of the case group.

The criteria for noninclusion for both cases and controls were metastasis, pregnant, or lactating women, hysterectomy and/or oophorectomy, presence of any cognitive, psychiatric or mobility difficulties, and upper and/or lower limb amputation. Those with a history of orthopedic, bone-metabolic or thyroid conditions, arthritis, arthrosis, autoimmune diseases, genetic syndromes, gastrointestinal diseases (i.e., celiac disease and inflammatory bowel diseases) and gastrectomy were also not included.

Those volunteers who had frailty fracture within 12 months prior to the interview were not included, as well as those using oral or injectable contraceptives for a period of more than 3 months in the last year, using corticosteroids, anticonvulsants, anticoagulants, and bisphosphonates for 3 consecutive months before the interview and using calcium and/or vitamin D supplements in the 6 months prior to data collection.

The study was approved by the Research Ethics Committee of the Hospital das Clínicas of the Federal University of Goiás, under opinion no. 751.387/2014 and amendment 1, no. 178.4248/2016. Participants were informed about the research procedures, as well as risks and benefits and those who agreed to participate signed the informed consent form.

### 2.2. Sample Size

The sample calculation was based on a study that had a higher prevalence of high bone mineral density in the lumbar spines evaluated by quartiles in women with breast cancer, since it is a risk for breast cancer [10].

A significance level of 95%, a test power (1 - *β*) of 80%, a ratio of two controls for each case and an odds ratios (OR) of 1.95 was considered clinically relevant, with a prevalence of high bone mineral density among the controls of 24.8% [10], and obtaining a minimum required sample of 120 cases and 239 controls.

For each newly diagnosed breast cancer patient, two controls were selected, matched by age (quinquennium), body mass index (BMI) [20], and menopausal status. Premenopausal refers to cases where the woman reported maintenance or absence of menstruation for less than 12 months, and postmenopausal refers to prior amenorrhea for a minimum period of 12 months, regardless of age [21].

### 2.3. Assessment of Bone Mineral Density

A standard questionnaire was used to collect socioeconomic, demographic, and clinical data. Bone mineral density (g/cm^2^) was assessed by the dual-energy x-ray absorptiometry (DXA) DPX NT device (General Electric Medical Systems Lunar®, Madison, USA). Volunteers were instructed not to perform other procedures with contrast or radiation the day before the interview. The exam was performed in the supine position, with bare feet, light clothes, and no metallic objects [22].

The anatomical sites evaluated included the lumbar spine (L1–L4), femoral neck, and total femur, which were segregated into quartiles (Q1 lower and Q4 highest) for total sample and menopausal status. The results obtained by densitometry were classified by the T-score for postmenopausal women, in which those with a T-score > -1 were classified as having normal BMD and those with a T-score ≤ -1.1 were classified as having low BMD (i.e., osteopenia or osteoporosis) [23, 24]. For premenopausal women, the Z-score was used instead of the T-score and were classified as being within the estimated value if their Z-score was > -2.00 and below the estimated value when it was ≤ -2.00 [25].

### 2.4. Assessment of Other Variables of Interest

The following parameters were assessed: height in meters, evaluated by stadiometer fixed to the wall; the total body weight in kilograms as assessed by DXA; the age in years; the BMI in kg/m^2^ classified as having no excess weight (adult: < 25 kg/m^2^ and elderly: < 27 kg/m^2^) and overweight (adult ≥ 25 kg/m^2^ and elderly ≥ 27 kg/m^2^) [20]; age of early menarche (≥ 12 years) and not precocious (< 12 years); late menopausal age (≥ 55 years) and not later (< 55 years); late age of first gestation (> 30 years) and not late (≤ 30 years); presence or absence of children; breastfed or not; family history in first degree of breast cancer [26]. In addition, we evaluated smoking (nonsmokers or previous/current smokers), alcohol consumption (not consuming or consuming), and physical activity level, classified as active (≥ 600 MET/min/wk) or sedentary (< 600 MET/min/wk) [27].

Skin color (white or nonwhite), marital status (with or without partner), education (< or ≥ 9 years of schooling), family income/month (< or ≥ three times the minimum wage), home city (Goiânia or other cities), relative skeletal muscle index in Kg/m^2^ [28], use of hormone replacement therapy (yes or no), and waist circumference (normal if < 80 or elevated if ≥ 80 cm) were investigated [20] and were used as an adjustment in the statistical analysis.

### 2.5. Statistical Analysis

The results of the continuous variables were presented as the means and standard deviation (SD). A Shapiro-Wilk normality test was performed, and for comparisons between groups, unpaired Student's t-test was used in the presence of normality and the Mann-Whitney test in the absence. Absolute and relative frequencies, n (%), were used for the categorical variables, and the Fisher's exact test was used to assess the degree of homogeneity or comparability between the groups.

Associations, by reason of cross products, were estimated for the total sample and stratified by menopausal status. Logistic regression was used to determine the breast cancer, and the crude and adjusted analyses were performed using the backward procedure. This analysis considered the sociodemographic, economic, behavioral, clinical, and body composition variables with* p* < 0.20 as eligible for the model. In addition, the values of this analysis are presented by an odds ratio (OR) with 95% confidence interval (95% CI). SPSS software v23 (IBM Corp., Chicago, IL, USA) was used, and a significance level of 5% adopted was for all tests.

## 3. Results

A total of 376 women participated, 142 in the case group and 234 in the control group, with 53.4% reporting postmenopausal status (201 total, 73 cases versus 128 controls). No differences were found in the total sample or by menopausal status when comparing the nutritional status measured by BMI, age at menarche, age at first gestation, presence or absence of children, breastfeeding or not, family history (first degree) of breast cancer, smoking and alcohol consumption (Table 1).

On the other hand, it was verified that the volunteers in the case group had a mean height (m) and total body weight (kg) lower than those in the control group for the total and premenopausal samples (*p* < 0.05). Cancer patients were more sedentary than their matched controls in the total (*p* = 0.005) and postmenopausal samples (*p* = 0.017). In relation to socioeconomic variables, income, and education, on average, the cases had less than three minimum wages per month (*p* < 0.001) and less than nine years of schooling (*p* < 0.05) (Table 1).

The BMD assessment revealed that in the total, pre- and postmenopausal samples, there were no differences between the groups. Except for the T-score that obtained a higher proportion of low BMD in the cases for the femoral neck (*p* = 0.032) (Table 2). When odds ratio (OR) was assessed, women allocated to the highest BMD quartile of the lumbar spine presented a higher chance of breast cancer than the first quartile (OR = 2.31;* p* = 0.045). No associations were found when menopausal status was assessed separately (Table 3).

The anatomical sites of the femoral neck and total femur were not associated in the adjusted model evaluated by the backward method. Therefore, these variables were withdrawn from the adjusted analysis (*p* > 0.20), justifying their absences in Table 3.

## 4. Discussion

This study revealed a positive association between the highest quartile of lumbar spine BMD and new breast cancer diagnosis in the total sample, but not between menopausal statuses. In addition, BMD of the femoral neck and total femur were not associated with breast cancer diagnosis.

These results agree with previous studies that revealed a higher frequency of breast cancer in women with high BMD [29–32]. In a cohort study conducted in Canada using data from January 1999 through December 2007 taken from the Canadian Cancer Registry, elevated lumbar spine BMD was an independent risk factor for any type of breast cancer in women aged 50 years or older [12].

In another study conducted between 1986 and 1988, which was the first prospective cohort conducted in the United States, the association between BMD and the risk of developing breast cancer in elderly women was assessed and the risk for neoplasia was found to be about of 30–50% higher for each increased standard deviation of BMD in the multivariate model. In that same study, women above the 25th percentile had a 2–2.5-fold higher risk of breast cancer than those in the lowest percentile at all sites evaluated [33].

In a case-control study similar to the present study, The Marburg Breast Cancer and Osteoporosis Trial (MABOT) II measured the BMD of the lumbar spine femoral neck and hip by the DXA method and quantitative ultrasonography in women who were pre- and postmenopausal with an incident breast cancer diagnosis without previous treatment, with the same measurements taken in their control group. All measurements showed significantly higher BMD values (*p* < 0.05) in cancer patients in both methods [31]. In our study, where the DXA method was used exclusively, there was an association between lumbar spine BMD and the outcome of mammary neoplasia, whereas in other anatomical sites no association was identified. In addition, menopausal status was assessed, and no significant difference of BMD was found between pre- and postmenopausal women.

Research suggests that the estrogen exposure during women's lives affects the relationship between BMD and mammary neoplasia. This is because the hormone plays a key role in the maintenance of bone mass [1–3]; on the other hand, estrogen is a strong marker of risk for breast cancer if there is long term exposure [4, 5]. Therefore, in this study we sought to evaluate BMD in groups of women in different age groups, with menopause considered as the cutoff point.

Factors such as early menarche, nulliparity, first pregnancy at a late age, short period or absence of breastfeeding, and late menopause are considered risk factors for the onset of breast cancer and reflect a prolonged exposure of estrogen during menacing [32, 34]. Likewise, BMD is a marker of estrogenic accumulation, due to the active participation of the hormone in the formation and resorption of bone during the reproductive age [35].

In addition, estrogen influences the production of cytokines and growth factors, such as insulin-like growth factor (IFG-1), interleukin-6 (IL-6), osteoprotegerin (OPG), and receptor activator of nuclear factor kappa-B ligand (RANKL), which are involved in bone turnover [1, 36, 37] and mammary cancer [38, 39].

Other factors have an effect on high levels of bone mass. Genetic factors are often mentioned, representing 75–80% of the peak bone mass variation [40], anthropometric and body composition tests in which excess body weight protects the bone by the production of estrogen and leptin (the hormone that participates in the regulation of the development of osteoclasts) by adipose tissue, as well as the overloading on the skeleton [41, 42]. Nutritional factors derived from the ingestion of macronutrients (protein with an osteoanabolic effect) and micronutrients (calcium and vitamin D) are also involved, as well as physical activity [42].

Obesity and a sedentary lifestyle pose a risk for pre- and postmenopausal development of breast cancer [43–46]. According to National Health and Nutrition Examination Survey (NHNES) cross-sectional study, the light physical activity was associated with increased BMD of about 3 mg/cm^3^ and reduced risk of osteoporosis in the spine in women over 50 years old [47]. Regarding the frequency of BMD assessed by T-score and Z-score in this study, the case group in the total and postmenopausal samples presented low BMD relative to the control and were also more sedentary, with no difference in relation to obesity.

One limitation of this study was the difficulty in composing the control group. In studies with a case-control design, it is recognized that the cases are imposed in the sample, according to the previously defined criteria. The control group was paired to participants in the case group with respect to age, menopausal status, and BMI. However, because of the newly diagnosed women who made up the case group, who represent a population coming from different regions, it was not possible to apply techniques for homogenization in relation to all variables of the study, such as sociodemographic factors. In addition, the variables used for the pairing prevented the selection of women without neoplasia and those in the same region of the study. However, it should be pointed out that our study is an innovative one for evaluating the association of BMD with breast cancer in newly diagnosed Brazilian women, applying a gold-standard evaluation method for bone densitometry.

## 5. Conclusion

The results suggest that highest quartile of lumbar spine BMD was positively associated with breast cancer in the total sample, when adjusted for confounding variables and compared to women in the lowest quartile. In relation to the neck of the femur and total femur, no such association was observed, as well as between pre- and postmenopausal groups.

## Figures and Tables

**Table 1:** Socioeconomic, clinical, behavioral, and anthropometric variables by menopausal status and breast cancer diagnosis. Goiânia, 2014–2017.

Variables	Total sample (n = 376)			Premenopausal (n = 175)			Postmenopausal (n = 201)		
	Cases	Controls	*p*	Cases	Controls	*p*	Cases	Controls	*p*
	n = 142	n = 234		n = 69	n = 106		n = 73	n = 128	
	Mean (SD)	Mean (SD)		Mean (SD)	Mean (SD)		Mean (SD)	Mean (SD)	
	n (%)	n (%)		n (%)	n (%)		n (%)	n (%)	

Height (m)	1.57 (±0.06)	1.59 (±0.07)	**0.005**	1.58 (±0.07)	1.61 (±0.06)	**0.006**	1.55 (±0.07)	1.57 (±0.07)	0.107

Total body weight (kg)	66.35 (±13.92)	69.06 (±13.86)	**0.029**	65.42 (±13.27)	70.46 (±14.81)	**0.016**	67.23 (±14.54)	67.91 (±12.97)	0.524

Age	50.72 (±11.33)	51.39 (±11.30)	0.536	41.84 (±6.43)	41.66 (±6.97)	0.751	59.11 (±8.10)	59.45 (±7.03)	0.753

Family income/ month									
<3 minimum salaries	110 (80.3)	114 (49.6)	**<0.001**	51 (76.1)	49 (47.1)	**<0.001**	59 (84.3)	65 (51.6)	**<0.001**
≥3 minimum salaries	27 (19.7)	116 (50.4)		16 (23.9)	55 (52.9)		11 (15.7)	61 (48.4)	

Education									
<9 years	82 (57.7)	78 (33.5)	**<0.001**	29 (42.0)	23 (21.7)	**0.006**	53 (72.6)	55 (43.3)	**<0.001**
≥9 years	60 (42.3)	155 (66.5)		40 (58.0)	83 (78.3)		20 (27.4)	72 (56.7)	

BMI (Kg/m^2^)									
Not overweight	67 (47.2)	89 (38.0)	0.085	35 (50.7)	41 (38.7)	0.122	32 (43.8)	48 (37.5)	0.454
Overweight	75 (52.8)	145 (62.0)		34 (49.3)	65 (61.3)		41 (56.2)	80 (62.5)	

Age of menarche									
≥12 years	113 (82.5)	189 (81.8)	>0.999	52 (76.5)	82 (77.4)	>0.999	61 (88.4)	107 (85.6)	0.664
<12 years	24 (17.5)	42 (18.2)		16 (23.5)	24 (22.6)		8 (11.6)	18 (14.4)	

Age of menopause									
<55 years							65 (90.3)	120 (95.2)	0.233
≥55 years							7 (9.7)	6 (4.8)	

	Mean (SD)	Mean (SD)		Mean (SD)	Mean (SD)		Mean (SD)	Mean (SD)	
	n (%)	n (%)		n (%)	n (%)		n (%)	n (%)	

Age of 1st gestation									
≤30 years	113 (90.4)	190 (92.7)	0.536	57 (89.1)	80 (92.0)	0.580	56 (91.8)	110 (93.2)	0.766
>30 years	12 (9.6)	15 (7.3)		7 (10.9)	7 (8.0)		5 (8.2)	8 (6.8)	

Children									
Yes	128 (99.2)	206 (99.5)	>0.999	65 (100.0)	87 (98.9)	>0.999	63 (98.4)	119 (100.00)	0.350
No	1 (0.8)	1 (0.5)		---	1 (1.1)		1 (1.6)	---	

Breast-feeding									
Yes	124 (94.7)	198 (93.4)	0.817	62 (93.9)	85 (93.4)	>0.999	62 (95.4)	113 (93.4)	0.750
No	7 (5.3)	14 (6.6)		4 (6.1)	6 (6.6)		3 (4.6)	8 (6.6)	

Family history (1st grade)									
No	121 (85.8)	209 (90.1)	0.243	64 (92.8)	98 (93.3)	>0.999	57 (79.2)	111 (87.4)	0.155
Yes	20 (14.2)	23 (9.9)		5 (7.2)	7 (6.7)		15 (20.8)	16 (12.6)	

Smoking									
No	216 (92.3)	124 (87.3)	0.147	69 (100.00)	106 (100.00)	---	55 (75.3)	110 (85.9)	0.084
Yes	18 (7.7)	18 (12.7)		---	---		18 (24.7)	18 (14.1)	

Alcohol consumption									
No	130 (91.5)	209 (89.3)	0.593	63 (91.3)	92 (86.6)	0.468	67 (91.8)	117 (91.4)	>0.999
Yes	12 (8.5)	25 (10.7)		6 (8.7)	14 (13.2)		6 (8.2)	11 (8.6)	

Physical activity (MET/min/wk)									
Active	65 (45.8)	142 (60.7)	**0.005**	31 (44.9)	59 (55.7)	0.216	34 (46.6)	82 (64.8)	**0.017**
Sedentary	77 (54.2)	92 (39.3)		38 (55.1)	47 (44.3)		39 (53.4)	45 (35.2)	

Continuous variables are presented as mean and standard deviation (SD). In the presence of normality, a Student's t-test was used, and in its absence the Mann-Whitney test was used. Categorical variables are in absolute and relative values n (%). Fisher's exact test was applied with a significance level of 5%. Missing values are ignored or uncollected values. BMI = body mass index; MET = metabolic equivalent of the task.

**Table 2:** Characterization of bone mineral density by menopausal status and diagnosis of breast cancer. Goiânia. Brazil. 2014–2017.

Variables	Total sample (n = 376)			Premenopausal (n = 175)			Postmenopausal (n = 201)		
	Cases	Controls	*p*	Cases	Controls	*p*	Cases	Controls	*p*
	n = 142	n = 234		n = 69	n = 106		n = 73	n = 128	
Lumbar spine. BMO (g/ cm^2^)									

Q1 (lower)	42 (29.6)	58 (24.8)	0.700	20 (29.0)	26 (24.5)	0.905	22 (30.1)	32 (25.0)	0.666
Q2	34 (23.9)	59 (25.2)		15 (21.7)	27 (25.5)		19 (26.0)	32 (25.0)	
Q3	37 (26.1)	60 (25.6)		18 (26.1)	28 (26.4)		19 (26.0)	32 (25.0)	
Q4 (highest)	29 (20.4)	57 (24.4)		16 (23.2)	25 (23.6)		13 (17.8)	32 (25.0)	
Z-score (≤ -2.0)				1 (1.4)	1 (0.9)	>0.999			
T-score (≤ -1.1)							44 (60.3)	69 (53.9)	0.460

Femoral neck. BMO (g/cm^2^)									

Q1 (lower)	55 (38.7)	59 (25.2)	0.051	24 (34.8)	26 (24.5)	0.428	31 (42.5)	33 (25.8)	0.100
Q2	31 (21.8)	59 (25.2)		19 (27.5)	28 (26.5)		12 (16.5)	31 (24.2)	
Q3	29 (20.4)	58 (24.8)		13 (18.8)	26 (24.5)		16 (21.9)	32 (25.0)	
Q4 (highest)	27 (19.0)	58 (24.8)		13 (18.8)	26 (24.5)		14 (19.2)	32 (25.0)	
Z-score (≤ -2.0)				3 (4.3)	1 (0.9)	0.302			
T-score (≤ -1.1)							8 (11.0)	4 (3.1)	**0.032**

Total femur. BMO (g/cm^2^)^a^									

Q1 (lower)	49 (36.8)	56 (25.6)	0.167	29 (44.6)	26 (26.3)	0.071	20 (29.4)	30 (25.0)	0.806
Q2	29 (21.8)	54 (24.7)		15 (23.1)	24 (24.2)		14 (20.6)	30 (25.0)	
Q3	28 (21.1)	55 (25.1)		9 (13.8)	25 (25.3)		19 (27.9)	30 (25.0)	
Q4 (highest)	27 (20.3)	54 (24.7)		12 (18.5)	24 (24.2)		15 (22.1)	30 (25.0)	
Z-score (≤ -2.0)				-* *-* *-	1 (1.0)	>0.999			
T-score (≤ -1.1)							5 (7.4)	2 (1.7)	0.101

Continuous variables are presented as mean and standard deviation (SD). In the presence of normality, the Student's t-test was used, and in its absence the Mann-Whitney test was used. The categorical variables are in absolute and relative values n (%). Fisher's exact test was applied with a significance level of 5%. Cut points were obtained by the quartile of bone mineral density (BMD) for lumbar spine, total femur, and femoral neck based on the control group of the total sample and stratified by menopausal status. Lumbar column in the total sample: Q1 (≤0.998), Q2 (0.999–1.103), Q3 (1.104–1.204), and Q4 (≥1.205); premenopausal: Q1 (≤1.091), Q2 (1.092–1.159), Q3 (1.160–1.250), and Q4 (≥1.251) and postmenopausal: Q1 (≤0.925), Q2 (0.926-1.030), Q3 (1.031-1.150), and Q4 (≥1.151). Femoral neck in the total sample: Q1 (≤0.860), Q2 (0.861–0.958), Q3 (0.959–1.044), and Q4 (≥1.045); premenopausal: Q1 (≤0.929), Q2 (0.930–1.010), Q3 (1.011–1.104), and Q4 (≥1.105) and postmenopausal: Q1 (≤0.839), Q2 (0.840–0.914), Q3 (0.915–0.992), and Q4 (≥0.993). Total femur in the total sample: Q1 (≤0.901), Q2 (0.902–0.985), Q3 (0.986–1.076) and Q4 (≥1.077); premenopausal: Q1 (≤0.943), Q2 (0.944–1.032), Q3 (1.033–1.125), and Q4 (≥1.126) and postmenopausal: Q1 (≤0.859), Q2 (0.860–0.948), Q3 (0.949–1.041), and Q4 (≥1.042). The sample consisted of 133 cases (65 pre- and 68 postmenopausal) and 219 controls (99 pre- and 120 postmenopausal).

**Table 3:** Odds Ratio (crude regression) between bone mineral density for menopausal status and breast cancer diagnosis. Goiânia. Brazil. 2014–2017.

	*Total sample *(n = 376)				*Premenopausal *(n = 175)				*Postmenopausal *(n = 201)			
	Not adjusted	*p*	Adjusted	*p*	Not adjusted	*p*	Adjusted	*p*	Not adjusted	*p*	Adjusted	*p*
	OR (IC95%)		OR (IC95%)		OR (IC95%)		OR (IC95%)		OR (IC95%)		OR (IC95%)	

*Lumbar spine (g/cm* ^*2*^)												
Q1 (lower)	1		1^a^		1		1^b^		1		1^c^	
Q2	0.83	0.540	1.51	0.285	0.72	0.458	2.79	0.140	0.86	0.71	1.73	0.380
	(0.46–1.50)		(0.71–3.23)		(0.31–1.70)		(0.71–10.91)		(0.39–1.89)	4	(0.51–5.94)	
Q3	0.86	0.606	1.79	0.144	0.84	0.672	2.85	0.105	0.86	0.71	3.67	0.053
	(0.48–1.54)		(0.82–3.93)		(0.36–1.92)		(0.80–10.12)		(0.39–1.89)	4	(0.98–13.76)	
Q4 (highest)	0.92	0.787	2.31	**0.045**	0.83	0.674	3.07	0.130	0.59	0.221	4.04	0.060
	(0.52–1.65)		(1.02–5.25)		(0.35–1.96)		(0.72–13.08)		(0.25–1.37)		(0.94–17.36)	

The values are presented by odds ratio (OR) and p-value with a confidence interval of 95% (95% CI) and a significance level of 5%. The adjusted models were performed by the backward method (*p* < 0.20), and the variables for the lumbar spine correspond with the letters in the table as follows: (a) family history of breast cancer (1st degree), age, use of hormone replacement therapy, city of residence, years of education, family income/month, body mass index, and height; (b) level of physical activity, marital status, city of residence, early menarche, family income/month, height, waist circumference, total body weight, body mass index, and number of children born; (c) hormone replacement therapy, age of first late gestation, city of residence, schooling, family income/month, height, waist circumference, and body mass index. Femoral neck and total femur were not associated with the backward method and were automatically withdrawn from the adjusted analysis (*p* > 0.20).

## Data Availability

The data used to support the findings of this study are available from the corresponding author upon request.

## Abbreviations

BMD: Bone mineral density
BMI: Body mass index
DXA: Dual-energy x-ray absorptiometry
OR: Odds ratios
SD: Standard deviation
MABOT: Marburg Breast Cancer and Osteoporosis Trial
IFG-1: Insulin-like growth factor
OP: Osteoprotegerin
RANKL: Nuclear factor-activating receptor kappa B ligand.
